# Perioperative neutrophil-to-lymphocyte ratio trajectories and creatinine-defined acute kidney disease after cardiac surgery: a study using the INSPIRE database

**DOI:** 10.3389/fcvm.2026.1952199

**Published:** 2026-09-08

**Authors:** Feifan Hu, Yefang Qian, Lanxin Hu, Duanqi Zhu, Yuzhi Jiang, Ke Ding, Xinglong Liu, Hongwei Shi, Lihai Chen, Hao Cheng, Yali Ge

**Affiliations:** 1 Department of Anesthesiology, Perioperative and Pain Medicine, Nanjing First Hospital, Nanjing Medical University, Nanjing, China; 2 Department of Affective Disorders II, Hebei Provincial Mental Health Center, The Sixth Clinical Medical College of Hebei University, Baoding, China; 3 Department of Anesthesiology and Pain Medicine, Geriatric Hospital of Nanjing Medical University, Nanjing, China

**Keywords:** acute kidney disease, cardiac surgery, group-based trajectory modeling, inflammation, neutrophil-to-lymphocyte ratio, persistent AKD

## Abstract

**Background:**

Acute kidney disease (AKD) encompasses persistent or newly developing renal dysfunction beyond 7 days after surgery. Associations between perioperative neutrophil-to-lymphocyte ratio (NLR) changes and AKD after cardiac surgery remain unclear. We identified NLR trajectories and examined their associations with overall, persistent, and delayed AKD.

**Methods:**

This retrospective, hypothesis-generating cohort study used the single-center INSPIRE database. Adults with NLR available in at least four of five perioperative windows were classified using group-based trajectory modeling. The primary outcome was AKD during postoperative days 8–90. Time-to-event, competing-risk, follow-up-weighted, early-AKI-stage, expanded-confounder, and medication-exposure sensitivity analyses were performed. Mortality and postoperative in-hospital hemodialysis/continuous renal replacement therapy (CRRT) records were exploratory secondary outcomes.

**Results:**

Of 2,265 patients assigned to a trajectory, 1,975 had day-8–90 creatinine follow-up; 298 developed AKD. Three data-driven trajectories were identified: low-response (T1), intermediate peak-recovery (T2), and high persistent-response (T3). Adjusted odds of overall AKD were higher for T2 vs. T1 (OR: 2.02, 95% CI: 1.35–3.04) and T3 vs. T2 (OR: 1.42, 95% CI: 1.02–1.96), with the clearest gradient for persistent AKD. Kaplan–Meier curves differed for overall and persistent AKD (both log-rank *P* < 0.001), and Cox and Fine–Gray analyses were concordant. Associations were broadly similar in sensitivity analyses, although adjustment for early AKI attenuated some adjacent contrasts. Ninety-day mortality and postoperative in-hospital hemodialysis/CRRT records were most frequent in T3, but these exploratory endpoints were uncommon and susceptible to residual confounding.

**Conclusions:**

Perioperative NLR trajectories were associated with creatinine-defined AKD after cardiac surgery, most clearly with persistent AKD. Concordant findings for mean perioperative and late postoperative NLR suggest that the magnitude and persistence of NLR elevation were more informative than relative within-patient variability.

## Highlights

- Three distinct perioperative NLR trajectories were identified, but they are single-center, data-driven classes that require external validation.
- Intermediate and high NLR trajectories were associated with higher odds of AKD, with the clearest graded association observed for persistent AKD.
- Mean perioperative and late postoperative NLR, but not relative within-patient variability, were consistently associated with AKD outcomes, highlighting the importance of the magnitude and persistence of perioperative inflammation.

## Introduction

Kidney dysfunction after cardiac surgery is usually evaluated as acute kidney injury (AKI) occurring within the first 7 postoperative days (1, 2). This early window captures the initial injury but not the subsequent course of kidney function, including recovery or newly developed dysfunction (3). Acute kidney disease (AKD) extends the assessment period to postoperative days 8–90, encompassing persistent as well as newly developed kidney dysfunction after the perioperative insult (4).

Inflammation contributes to both kidney injury and repair after cardiac surgery (5, 6). Cardiopulmonary bypass, ischemia–reperfusion, hemodynamic instability, oxidative stress, and postoperative complications can provoke a systemic inflammatory response that persists beyond the immediate perioperative period (3, 7). The neutrophil-to-lymphocyte ratio (NLR) is an inexpensive, routinely available marker that reflects the balance between neutrophil-mediated inflammation and lymphocyte-related immune regulation (8). However, most perioperative studies have examined NLR at a single time point, providing little information about how it changes after surgery (9, 10).

The systemic inflammatory response to cardiac surgery varies considerably among patients (11). Perioperative NLR may remain relatively low, rise transiently before declining, or remain elevated into the late postoperative period (12, 13). Group-based trajectory modeling can identify such longitudinal patterns without relying on prespecified cutoffs (14, 15). Applied to serial NLR measurements, this approach can show when and for how long NLR remains elevated, which cannot be determined from a single measurement. We therefore identified perioperative NLR trajectories in adults undergoing cardiac surgery and examined their associations with AKD during postoperative days 8–90. We further evaluated whether these associations differed between persistent and delayed AKD and compared trajectory-based findings with selected NLR summary metrics.

## Materials and methods

### Data source and study design

This retrospective cohort study used the INformative Surgical Patient dataset for Innovative Research Environment (INSPIRE) v1.4.2, a publicly available perioperative database derived from surgical cases at Seoul National University Hospital between 2011 and 2020 (16–18). We examined adults undergoing cardiac surgery to determine whether perioperative NLR trajectories were associated with postoperative AKD.

The original INSPIRE dataset was approved by the Institutional Review Board of Seoul National University Hospital (IRB No. H-2210-078-1368), which waived the requirement for informed consent because of the retrospective design; public release of the dataset was approved by the Institutional Data Review Board of Seoul National University Hospital (BD-R-2022-11-02) (16). The present analysis used only de-identified data.

### Study population

Adult patients undergoing cardiac surgery were eligible for inclusion. Patients were excluded if they had preoperative dialysis dependence, end-stage kidney disease, or extremely high baseline serum creatinine, defined as serum creatinine ≥4.0 mg/dL, because postoperative AKD could not be reliably ascertained using creatinine-based criteria in these patients. Patients entered the NLR-eligible trajectory cohort if they were aged ≥18 years, had a recorded eligible cardiac surgical procedure, and had NLR measurements in at least four of the five prespecified perioperative windows. Cardiac surgery was identified according to procedure records in the INSPIRE database and categorized as coronary artery bypass grafting, valve surgery, or other cardiac or great-vessel procedures. Group-based trajectory modeling was performed in this NLR-eligible cohort irrespective of subsequent AKD ascertainment. Outcome analyses were restricted to patients with at least one serum creatinine measurement during postoperative days 8–90. Patients without creatinine follow-up during this period were excluded from the primary AKD outcome analyses.

### NLR measurements and perioperative inflammatory variables

NLR was calculated by dividing the neutrophil count by the lymphocyte count (10). Five perioperative NLR windows were defined: the latest available measurement within 7 days before surgery and four postoperative windows (0–24, 24–48, 48–72, and 72–168 h). When multiple NLR measurements were available within a postoperative window, the median of the available values was used. NLR values were not imputed, and an observed measurement was required in at least four of the five windows for trajectory modeling. Window-specific availability is summarized in Supplementary Table S1.

To aid interpretation of the trajectory findings, we calculated four summary measures from the same perioperative windows. These metrics included mean perioperative NLR, late postoperative NLR, NLR standard deviation (SD), and NLR coefficient of variation (CV). Late postoperative NLR was defined as the NLR value in the 72–168 h postoperative window. NLR SD and CV represented absolute and relative within-patient variability, respectively. Mean NLR, late postoperative NLR, and NLR SD were log2-transformed and then standardized. NLR CV was standardized without log transformation. All summary metrics were analyzed per 1-SD increase and were calculated from observed values without imputation.

### Group-based trajectory modeling

Group-based trajectory modeling was used to identify perioperative NLR patterns across the five predefined windows (11, 19). Models with one to six classes and linear or quadratic time terms were evaluated using log2-transformed NLR. Patients were assigned to the class with the highest posterior probability. Selection considered Bayesian information criterion, a minimum class proportion of 5%, average posterior probability (AvePP) ≥0.70 for every class, separation of patterns, and clinical coherence (20); candidate-model statistics are reported in Supplementary Table S2. In addition to class-specific AvePP and size, we summarized the median, interquartile range, and minimum maximum posterior probability; the proportions with maximum posterior probability ≥0.70 and ≥0.90; and normalized posterior entropy. The final model was selected before outcome modeling. Outcome analyses used the maximum-posterior-probability class assignment and did not propagate classification uncertainty. Internal reproducibility of the selected three-class quadratic GBTM was assessed using five repetitions of five-fold cross-validation. In each split, the model was refitted in 80% of patients and applied to the held-out 20%. Fold-specific classes were aligned to the full-cohort trajectories according to NLR trajectory shape without reference to AKD outcomes. Held-out classification agreement, posterior probabilities, trajectory similarity, and repeated subject-level consistency were summarized.

### Outcomes

The primary outcome was creatinine-defined acute kidney disease (AKD) documented during postoperative days 8–90 (21). Baseline serum creatinine was defined as the most recent preoperative measurement within 7 days before surgery. AKD was staged according to serum creatinine criteria: stage 1, an increase of ≥0.3 mg/dL or 1.5–1.9 times baseline; stage 2, 2.0–2.9 times baseline; and stage 3, ≥3.0 times baseline or an absolute serum creatinine ≥4.0 mg/dL. For patients with multiple serum creatinine measurements during postoperative days 8–90, AKD severity was defined by the highest stage observed during this period. Overall AKD was defined as stage 1–3 AKD, and stage 2–3 AKD was analyzed as a severity-based secondary outcome (22). To characterize ascertainment support, we also summarized, among AKD cases, the number of day-8–90 creatinine measurements and the number of values independently meeting AKD criteria.

Early postoperative acute kidney injury (AKI) was defined within the first 7 postoperative days using serum creatinine-based Kidney Disease: Improving Global Outcomes (KDIGO) criteria: an increase of ≥0.3 mg/dL within 48 h or ≥1.5 times baseline within 7 days (22). Persistent AKD was defined as AKD during postoperative days 8–90 among patients with early postoperative AKI, whereas delayed AKD was defined as AKD during postoperative days 8–90 among patients without early postoperative AKI (23).

### Exploratory outcomes

Exploratory secondary outcomes were 28-, 30-, and 90-day all-cause mortality, in-hospital mortality, and positive postoperative inpatient ward records for hemodialysis or continuous renal replacement therapy (CRRT). Mortality was determined from recorded dates. Dialysis/CRRT observations were mapped to the index operation by postoperative time; because the source was an inpatient ward table, this endpoint reflects postoperative in-hospital treatment recorded within 90 days and cannot establish complete post-discharge renal-replacement-therapy status.

### Covariates

Covariates were selected *a priori* based on clinical relevance and prior literature. The primary adjusted model included age, sex, body mass index, American Society of Anesthesiologists (ASA) physical status, emergency surgery, baseline serum creatinine, hypertension, diabetes, heart failure, myocardial infarction, surgery type, operation duration, and cardiopulmonary bypass duration (24). Two expanded sensitivity models additionally incorporated available perioperative factors. The first added mean intraoperative arterial pressure, any intraoperative vasoactive-drug use, and any perioperative transfusion. The second further added inpatient preoperative renin–angiotensin–aldosterone system inhibitor and diuretic records within 7 days, postoperative vasoactive-drug and candidate-nephrotoxin records during days 0–7, and systemic corticosteroid records from 1 day before through postoperative day 7. These expanded models were considered sensitivity analyses because postoperative exposures may reflect illness severity or lie on the pathway between surgery, NLR, and AKD. Outpatient medication history, infection, and several postoperative complications were unavailable.

### Missing data

Missing covariate data were handled using multiple imputation by chained equations, with 20 imputed datasets generated (25). Only covariates were imputed; NLR measurements, AKD status, AKD stage, AKD subtype, and time-to-event variables were not imputed. Estimates from adjusted regression models were pooled across imputed datasets using Rubin's rules.

### Statistical analysis

Continuous variables were summarized as median [interquartile range], and categorical variables as number (percentage). Baseline characteristics were summarized by NLR trajectory. To assess potential surveillance selection, patients with and without day-8–90 creatinine follow-up were compared using standardized mean differences (SMDs), with hypothesis-test *P* values reported descriptively.

Associations between NLR trajectories and AKD outcomes were assessed using unadjusted and fully adjusted models. The primary comparisons were adjacent trajectory contrasts between consecutively ordered trajectory groups. Logistic regression was used for overall AKD and stage 2–3 AKD. Ordinal logistic regression was used for AKD stage. Multinomial logistic regression was used for AKD subtypes, with no AKD as the reference category.

Because AKD ascertainment required clinically obtained creatinine measurements during postoperative days 8–90, inverse probability weighting (IPW) was used to address measured determinants of follow-up availability (26). The probability of AKD ascertainment was estimated in the full trajectory cohort using baseline demographic characteristics, comorbidities, surgical factors, early postoperative AKI, and trajectory group. Stabilized weights were truncated at the 1st and 99th percentiles. We report predicted-probability and weight distributions and effective sample sizes before and after truncation. Weighted logistic models used the same covariate set as the primary analyses (27).

Each NLR summary measure was analyzed separately using the primary covariate set. Kaplan–Meier curves and log-rank tests described cumulative occurrence of overall, persistent, and delayed AKD from postoperative day 8. Cox models incorporated time to the first qualifying creatinine measurement; patients were censored at death, the last available creatinine measurement, or day 90. A Fine–Gray model for overall AKD used exact recorded mortality time, with death before AKD treated as a competing event (28). Sensitivity analyses added postoperative-day-0–7 AKI stage, measured hemodynamic/transfusion variables, and medication-exposure variables separately to the primary model. Because early AKI and postoperative exposures temporally overlapped NLR trajectory definition, these models were not interpreted as causal mediation analyses. As a *post-hoc* exploratory analysis, late postoperative NLR (72–168 h) was evaluated as a simple threshold for overall AKD, persistent AKD among patients with early AKI, and delayed AKD among patients without early AKI. AUCs and Youden thresholds were estimated, and threshold stability was assessed using 1,000 bootstrap resamples with out-of-bag evaluation.

Exploratory patient-centered outcomes were analyzed in the full trajectory cohort. Given the relatively low event counts, adjusted associations were estimated using bias-reduced logistic regression with a parsimonious adjustment set comprising age, sex, ASA physical status ≥3, emergency surgery, baseline serum creatinine, and surgery type. All statistical tests were two-sided, and *P* < 0.05 was considered statistically significant. Analyses were performed using R version 4.5.1 (R Foundation for Statistical Computing, Vienna, Austria).

## Results

### Study population and perioperative NLR trajectories

**Figure 1:**
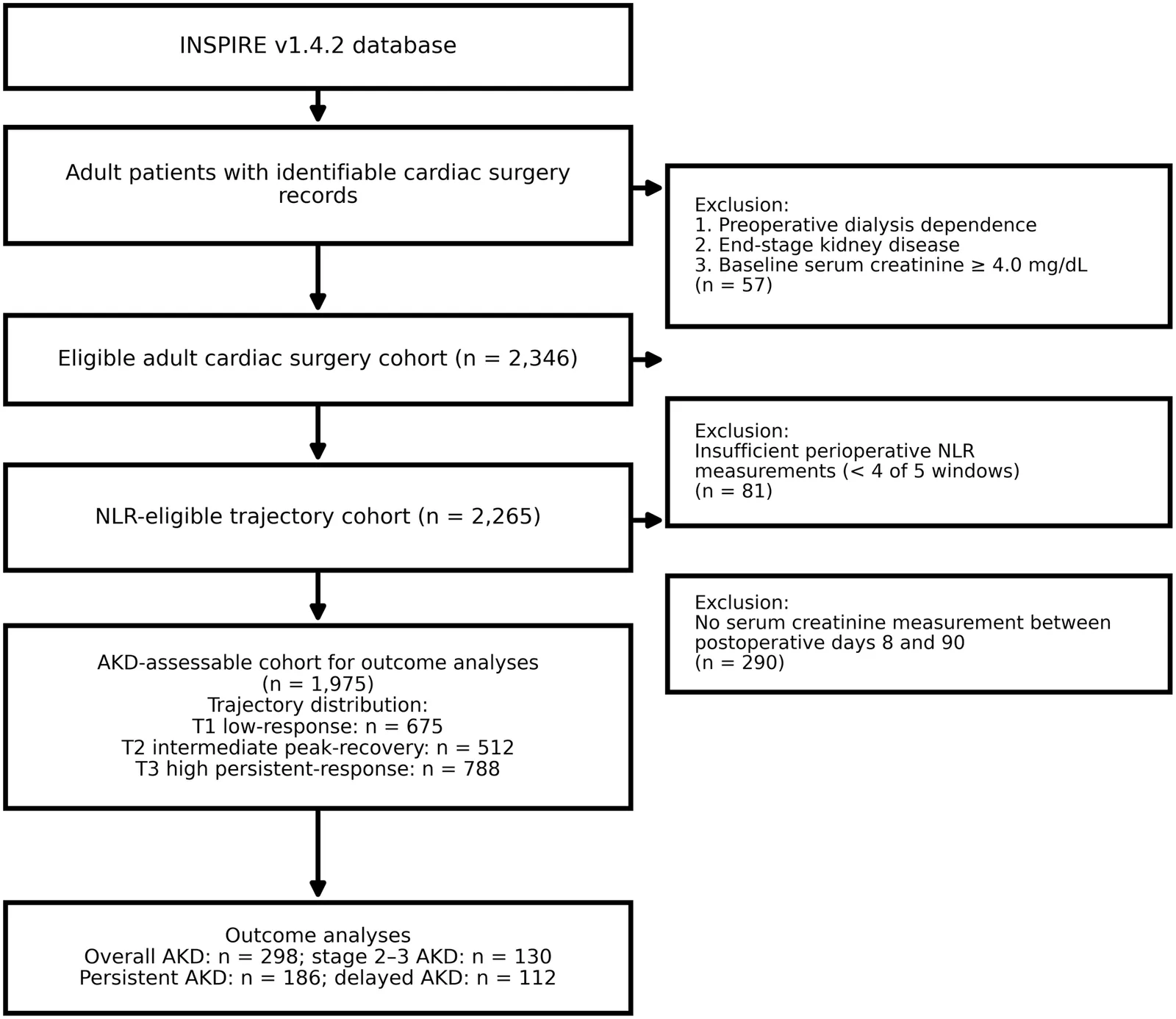
Study flowchart. Patients with preoperative dialysis dependence, end-stage kidney disease, or baseline serum creatinine ≥4.0 mg/dL were excluded before formation of the eligible adult cardiac surgery cohort. Patients with NLR measurements available in at least four of the five predefined perioperative windows were included in the NLR-eligible trajectory cohort for group-based trajectory modeling. AKD outcome analyses were subsequently restricted to the AKD-assessable cohort, defined as patients with at least one serum creatinine measurement between postoperative days 8 and 90. AKD, acute kidney disease; NLR, neutrophil-to-lymphocyte ratio.

Among 2,346 adults undergoing cardiac surgery, 2,265 had NLR measurements in at least four of five windows and were assigned to a trajectory. Of these, 1,975 had serum creatinine follow-up during postoperative days 8–90 and 290 did not. Compared with patients without follow-up, included patients were older (median 65 vs. 55 years; SMD 0.620), more often underwent coronary artery bypass grafting (38.7% vs. 3.1%; SMD 0.974), and had higher late postoperative NLR (median 6.42 vs. 4.31; SMD 0.556) (Supplementary Table S3). AKD occurred in 298 patients, including 130 with stage 2–3 AKD; 186 cases were persistent and 112 delayed. Among AKD cases, the median number of day-8–90 creatinine measurements was 17 [7–39]; 294 (98.7%) had at least two measurements, 213 (71.5%) had at least two qualifying values, and 85 (28.5%) were identified by a single qualifying value (Supplementary Table S4). The study selection process is shown in Figure 1.

**Figure 2:**
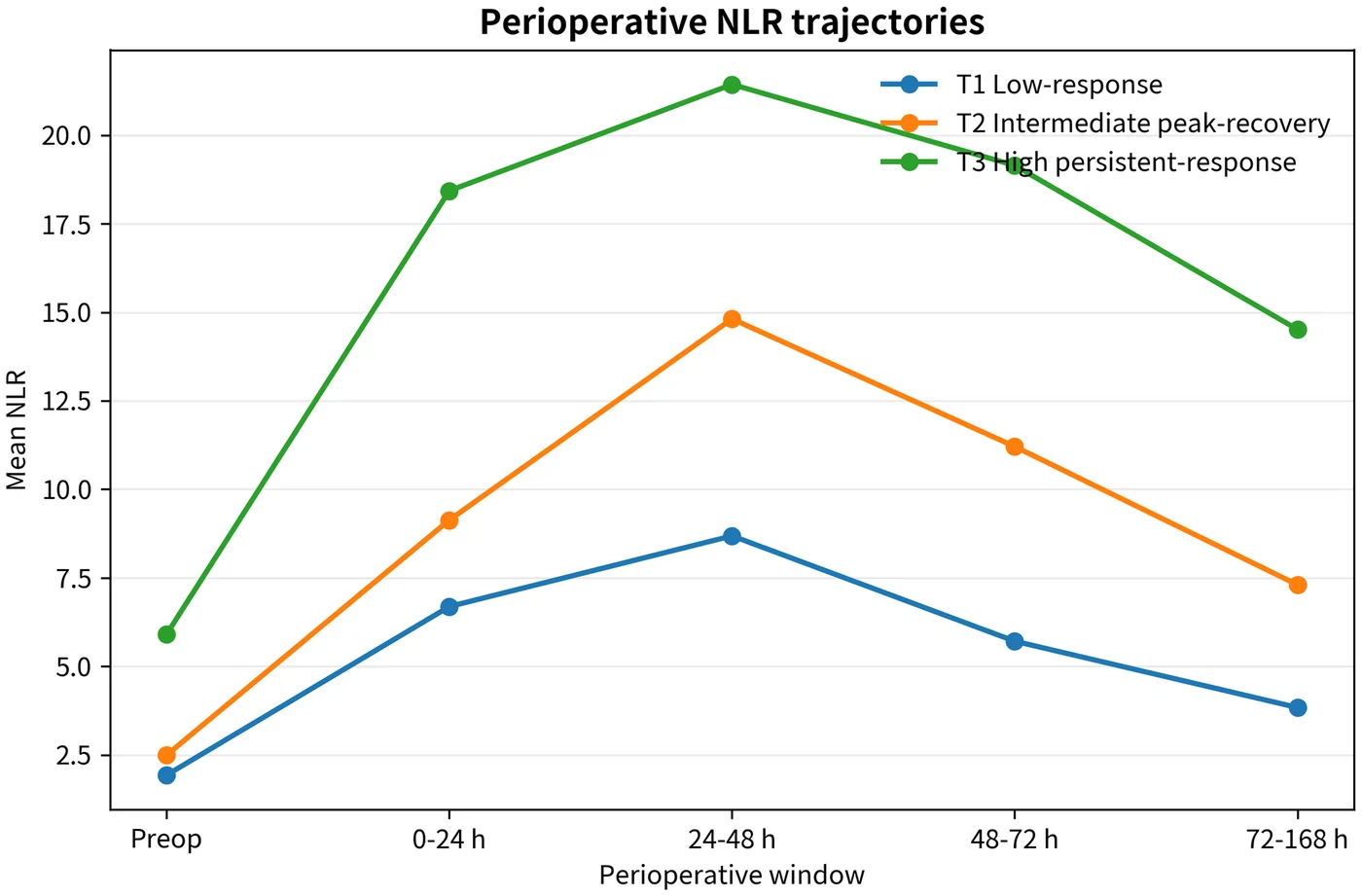
Perioperative NLR trajectories identified by group-based trajectory modeling. Three perioperative NLR trajectories were identified: T1, low-response; T2, intermediate peak-recovery; and T3, high persistent-response. Values are shown across the five predefined perioperative windows. NLR, neutrophil-to-lymphocyte ratio.

NLR window coverage and candidate-model statistics are summarized in Supplementary Tables S1, S2. Although the four-, five-, and six-class quadratic models had lower Bayesian information criterion values (27,711.4, 27,506.1, and 27,549.5, respectively) than the three-class quadratic model (27,856.3), their minimum class-specific AvePP values were 0.619, 0.610, and 0.471 and therefore did not meet the prespecified classification criterion. The three-class quadratic model was the best-fitting candidate for which every class had AvePP ≥0.70 and a proportion >5%. It identified low-response (T1), intermediate peak-recovery (T2), and high persistent-response (T3) trajectories (Figure 2), comprising 799, 588, and 878 patients. Baseline characteristics according to NLR trajectory in the AKD-assessable cohort are presented in Table 1. Mean maximum posterior probabilities were 0.880, 0.731, and 0.895; medians were 0.963, 0.742, and 0.983; and 83.0%, 62.9%, and 85.5% had maximum posterior probability ≥0.70, respectively (Supplementary Table S5). NLR peaked at 24–48 h in all groups. T1 had the lowest preoperative and postoperative levels; T2 showed an intermediate peak followed by recovery; and T3 began higher and remained elevated through 72–168 h (Supplementary Table S6).

**Table 1:** Baseline characteristics according to perioperative NLR trajectories in the AKD-assessable cohort.

Characteristic	Overall (*n* = 1,975)	T1 low-response (*n* = 675)	T2 intermediate peak-recovery (*n* = 512)	T3 high persistent-response (*n* = 788)	*P* value
Age, yr	65 [55, 75]	65 [55, 70]	65 [60, 75]	70 [60, 75]	<0.001
BMI, kg/m^2^	23.4 [20.8, 25.7]	23.9 [21.7, 26.0]	23.9 [21.5, 25.7]	22.2 [20.0, 25.0]	<0.001
Baseline creatinine, mg/dL	0.88 [0.74, 1.15]	0.84 [0.74, 1.05]	0.88 [0.77, 1.15]	0.92 [0.77, 1.15]	<0.001
Operation duration, min	350 [285, 420]	325 [260, 380]	358 [300, 420]	380 [290, 475]	<0.001
CPB duration, min	200 [155, 245]	175 [140, 220]	202 [160, 245]	210 [165, 260]	<0.001
Preoperative NLR	2.3 [1.6, 3.6]	1.7 [1.3, 2.3]	2.4 [1.8, 3.1]	3.6 [2.2, 6.6]	<0.001
Mean perioperative NLR	8.7 [6.0, 13.3]	5.3 [4.4, 6.2]	8.6 [7.6, 10.0]	14.9 [11.8, 19.6]	<0.001
Late postoperative NLR	6.4 [4.1, 11.7]	3.7 [2.9, 4.6]	6.8 [5.6, 8.9]	12.0 [7.5, 23.5]	<0.001
Female sex	742 (37.6%)	234 (34.7%)	176 (34.4%)	332 (42.1%)	0.003
Emergency surgery	217 (11.0%)	49 (7.3%)	41 (8.0%)	127 (16.1%)	<0.001
Hypertension	321 (16.9%)	114 (17.9%)	90 (18.1%)	117 (15.4%)	0.333
Diabetes	543 (28.6%)	215 (33.7%)	160 (32.2%)	168 (22.1%)	<0.001
Heart failure	299 (15.8%)	46 (7.2%)	82 (16.5%)	171 (22.5%)	<0.001
Myocardial infarction	176 (9.3%)	54 (8.5%)	55 (11.1%)	67 (8.8%)	0.274
ASA physical status					<0.001
I	57 (2.9%)	22 (3.3%)	14 (2.8%)	21 (2.7%)	
II	497 (25.5%)	217 (32.3%)	134 (26.5%)	146 (18.9%)	
III	1,283 (65.8%)	409 (60.9%)	333 (65.8%)	541 (70.1%)	
IV	112 (5.7%)	24 (3.6%)	25 (4.9%)	63 (8.2%)	
V	1 (0.1%)	0 (0.0%)	0 (0.0%)	1 (0.1%)	
Surgery type					<0.001
CABG	765 (38.7%)	367 (54.4%)	231 (45.1%)	167 (21.2%)	
Other cardiac/great vessel	150 (7.6%)	52 (7.7%)	26 (5.1%)	72 (9.1%)	
Valve	1,060 (53.7%)	256 (37.9%)	255 (49.8%)	549 (69.7%)	

Values are median [interquartile range] or *n* (%). *P* values compare distributions across the three trajectories in the AKD-assessable cohort. Percentages were calculated using available data for each variable. Trajectory membership was assigned using the final group-based trajectory model fitted in the NLR-eligible trajectory cohort. AKD, acute kidney disease; ASA, American Society of Anesthesiologists; BMI, body mass index; CABG, coronary artery bypass grafting; CPB, cardiopulmonary bypass; IQR, interquartile range; NLR, neutrophil-to-lymphocyte ratio.

### NLR trajectories and AKD outcomes

**Figure 3:**
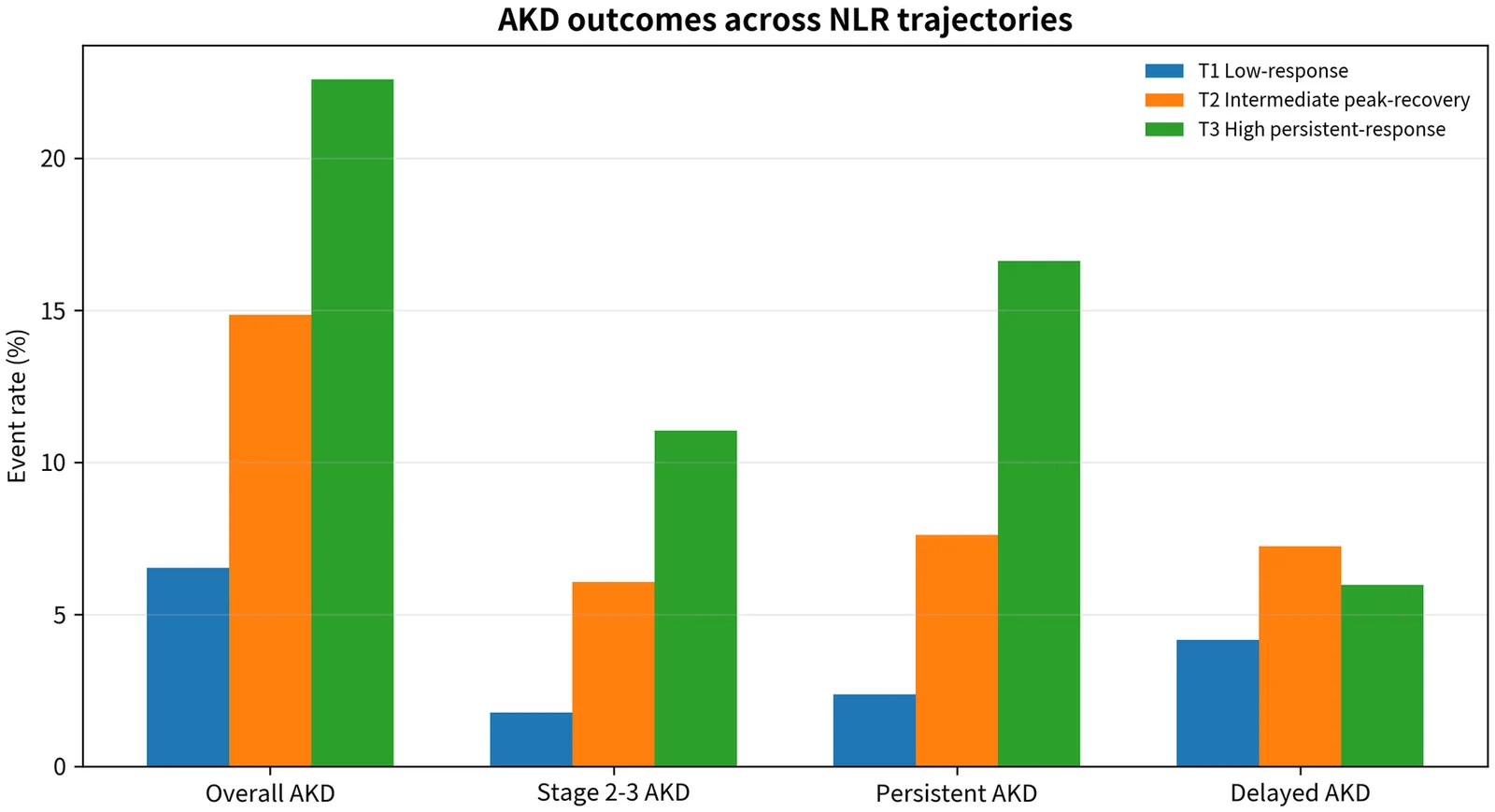
Proportions of AKD outcomes across perioperative NLR trajectories. Observed proportions of overall AKD, stage 2–3 AKD, persistent AKD, and delayed AKD are shown across the three NLR trajectories in the AKD-assessable cohort. AKD, acute kidney disease; NLR, neutrophil-to-lymphocyte ratio.

In unadjusted analyses, higher NLR trajectories were associated with greater odds of overall AKD and more severe AKD (Table 2). Compared with the low-response trajectory, the intermediate peak-recovery trajectory showed higher odds of overall AKD and stage 2–3 AKD. Compared with the intermediate peak-recovery trajectory, the high persistent-response trajectory showed a further increase in overall AKD risk, with a directionally positive association also observed for AKD severity (Figure 3).

**Table 2:** Associations of perioperative NLR trajectories with AKD and AKD severity.

Outcome	Contrast	Unadjusted OR (95% CI)	*P* value	Adjusted OR (95% CI)	*P* value
Overall AKD	T2 vs. T1	2.50 (1.69–3.70)	<0.001	2.02 (1.35–3.04)	<0.001
Overall AKD	T3 vs. T2	1.67 (1.25–2.25)	<0.001	1.42 (1.02–1.96)	0.035
Stage 2–3 AKD	T2 vs. T1	3.56 (1.81–7.00)	<0.001	2.67 (1.33–5.36)	0.006
Stage 2–3 AKD	T3 vs. T2	1.93 (1.26–2.95)	0.003	1.42 (0.89–2.27)	0.138
AKD stage (ordinal)	T2 vs. T1	2.53 (1.72–3.75)	<0.001	2.03 (1.35–3.05)	<0.001
AKD stage (ordinal)	T3 vs. T2	1.69 (1.26–2.26)	<0.001	1.41 (1.02–1.94)	0.037

Unadjusted estimates were obtained from univariable logistic regression for overall AKD and stage 2–3 AKD and univariable ordinal logistic regression for AKD stage. Adjusted estimates were obtained from multivariable models after multiple imputation for missing covariates and were pooled across 20 imputed datasets using Rubin's rules. The fully adjusted model included age, sex, body mass index, American Society of Anesthesiologists physical status, emergency surgery, baseline serum creatinine, hypertension, diabetes, heart failure, myocardial infarction, surgery type, operation duration, and cardiopulmonary bypass duration. AKD, acute kidney disease; CI, confidence interval; NLR, neutrophil-to-lymphocyte ratio; OR, odds ratio.

After adjustment for age, sex, body mass index, ASA physical status, emergency surgery, baseline serum creatinine, hypertension, diabetes, heart failure, myocardial infarction, surgery type, operation duration, and cardiopulmonary bypass duration, the associations remained generally consistent. Compared with the low-response trajectory, the intermediate peak-recovery trajectory was associated with higher odds of overall AKD (OR: 2.02, 95% CI: 1.35–3.04; *P* < 0.001) and stage 2–3 AKD (OR: 2.67, 95% CI: 1.33–5.36; *P* = 0.006). Compared with the intermediate peak-recovery trajectory, the high persistent-response trajectory was associated with higher odds of overall AKD (OR: 1.42, 95% CI: 1.02–1.96; *P* = 0.035), whereas the association with stage 2–3 AKD was directionally positive but did not reach statistical significance (OR: 1.42, 95% CI: 0.89–2.27; *P* = 0.138).

**Figure 4:**
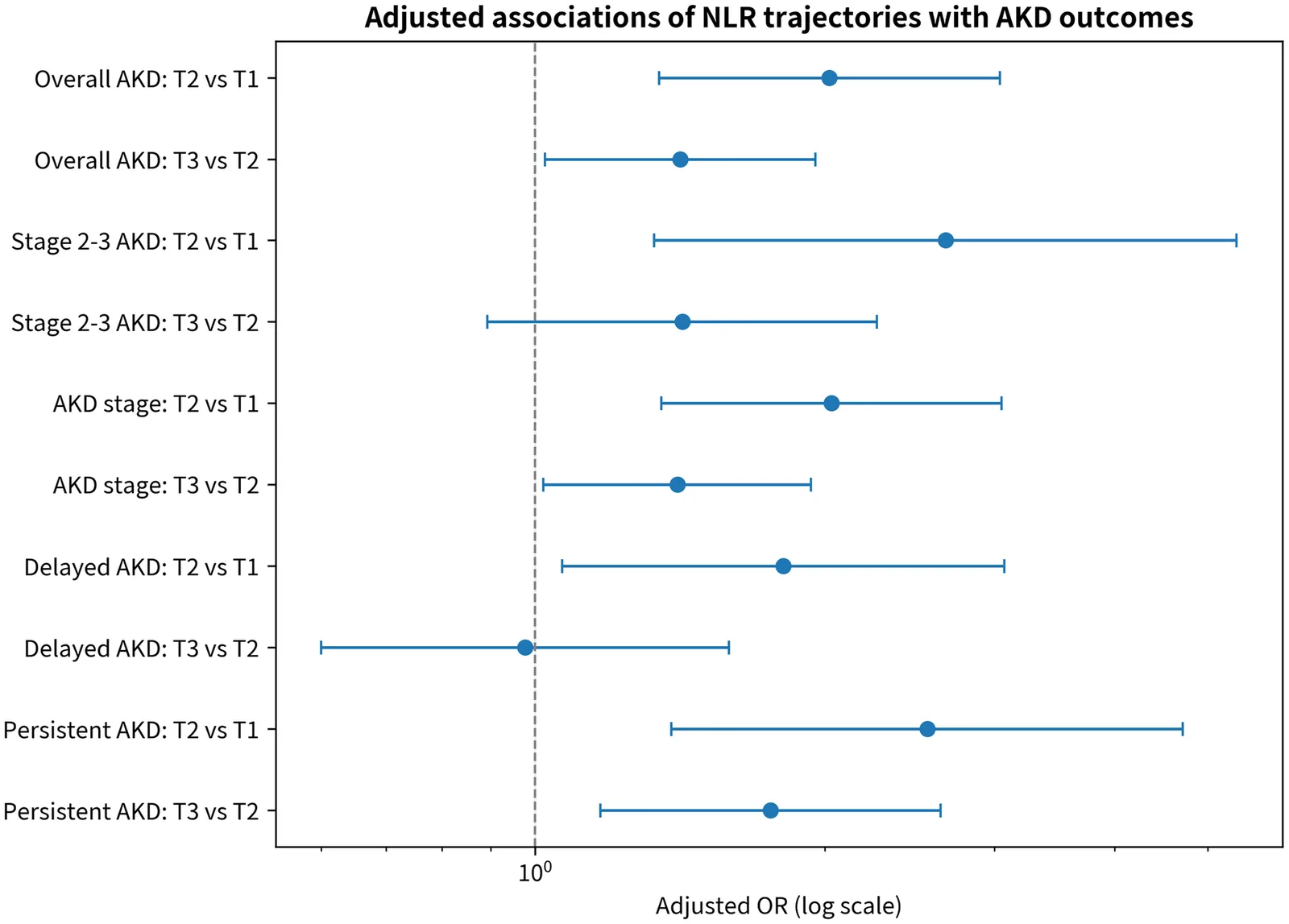
Adjusted associations of NLR trajectories with AKD outcomes. Forest plot showing adjusted odds ratios for adjacent trajectory contrasts. Points indicate odds ratios, and horizontal lines indicate 95% confidence intervals. Models were adjusted for the full covariate set described in the Methods. AKD, acute kidney disease; CI, confidence interval; NLR, neutrophil-to-lymphocyte ratio; OR, odds ratio.

Ordinal logistic regression showed a similar graded pattern for AKD stage. In the fully adjusted model, the intermediate peak-recovery trajectory was associated with higher AKD stage compared with the low-response trajectory (OR: 2.03, 95% CI: 1.35–3.05; *P* < 0.001), and the high persistent-response trajectory was associated with higher AKD stage compared with the intermediate peak-recovery trajectory (OR: 1.41, 95% CI: 1.02–1.94; *P* = 0.037). The fully adjusted associations between NLR trajectories and AKD outcomes are summarized in Figure 4.

The pattern across trajectories was more pronounced for persistent AKD than for delayed AKD (Table 3). For persistent AKD, the intermediate peak-recovery trajectory was associated with higher odds than the low-response trajectory (OR: 2.55, 95% CI: 1.39–4.71; *P* = 0.003), and the high persistent-response trajectory showed a further increase compared with the intermediate peak-recovery trajectory (OR: 1.76, 95% CI: 1.17–2.64; *P* = 0.007). The association with delayed AKD was directionally similar but weaker. The intermediate peak-recovery trajectory was associated with delayed AKD compared with the low-response trajectory (OR: 1.81, 95% CI: 1.07–3.07; *P* = 0.028), whereas the high persistent-response trajectory did not show further increase compared with the intermediate peak-recovery trajectory (OR: 0.98, 95% CI: 0.60–1.59; *P* = 0.924).

**Table 3:** Associations of perioperative NLR trajectories with AKD subtypes.

AKD subtype	Contrast	Adjusted OR (95% CI)	*P* value
Delayed AKD	T2 vs. T1	1.81 (1.07–3.07)	0.028
Delayed AKD	T3 vs. T2	0.98 (0.60–1.59)	0.924
Persistent AKD	T2 vs. T1	2.55 (1.39–4.71)	0.003
Persistent AKD	T3 vs. T2	1.76 (1.17–2.64)	0.007

Multinomial logistic regression was performed with no AKD as the reference outcome category. Models were adjusted for age, sex, BMI, ASA physical status, emergency surgery, baseline creatinine, hypertension, diabetes, heart failure, myocardial infarction, surgery type, operation duration, and CPB duration. AKD, acute kidney disease; ASA, American Society of Anesthesiologists; BMI, body mass index; CPB, cardiopulmonary bypass; OR, odds ratio; CI, confidence interval.

In the IPW sensitivity analysis, the truncated stabilized weights ranged from 0.881 to 1.712 and the effective sample size was 1,916.9, compared with 1,975 unweighted observations (Supplementary Table S7). The primary associations were largely unchanged: for overall AKD, the adjusted OR was 2.10 (95% CI: 1.36–3.24) for T2 vs. T1 and 1.43 (95% CI: 1.03–1.99) for T3 vs. T2 (Supplementary Table S8).

### Selected NLR summary metrics

Analyses of selected NLR summary metrics supported the trajectory-based interpretation (Table 4). Mean perioperative and late postoperative NLR were associated with overall AKD, stage 2–3 AKD, and persistent AKD. NLR SD showed smaller and less consistent associations, whereas NLR CV was not associated with any AKD outcome.

**Table 4:** Selected NLR summary metrics and AKD outcomes.

NLR metric (per 1 SD)	Outcome	Adjusted OR (95% CI)	*P* value
Mean perioperative NLR	Overall AKD	1.54 (1.32–1.81)	<0.001
Late postoperative NLR	Overall AKD	1.85 (1.58–2.17)	<0.001
NLR SD	Overall AKD	1.30 (1.11–1.52)	<0.001
NLR CV	Overall AKD	0.92 (0.79–1.06)	0.240
Mean perioperative NLR	Stage 2–3 AKD	1.45 (1.16–1.82)	0.001
Late postoperative NLR	Stage 2–3 AKD	1.88 (1.51–2.34)	<0.001
NLR SD	Stage 2–3 AKD	1.24 (0.99–1.55)	0.066
NLR CV	Stage 2–3 AKD	0.87 (0.70–1.09)	0.223
Mean perioperative NLR	Persistent AKD	1.62 (1.34–1.97)	<0.001
Late postoperative NLR	Persistent AKD	1.95 (1.61–2.35)	<0.001
NLR SD	Persistent AKD	1.40 (1.15–1.70)	<0.001
NLR CV	Persistent AKD	0.90 (0.75–1.08)	0.270
Mean perioperative NLR	Delayed AKD	1.29 (1.01–1.63)	0.038
Late postoperative NLR	Delayed AKD	1.45 (1.14–1.83)	0.002
NLR SD	Delayed AKD	1.12 (0.89–1.41)	0.347
NLR CV	Delayed AKD	0.97 (0.77–1.21)	0.767

Each NLR metric was standardized; estimates represent adjusted ORs per 1-SD increment. Mean NLR, late postoperative NLR, and NLR SD were log2-transformed and standardized; NLR CV was standardized without log transformation. Late postoperative NLR refers to the 72–168 h window. AKD, acute kidney disease; CI, confidence interval; CV, coefficient of variation; NLR, neutrophil-to-lymphocyte ratio; OR, odds ratio; SD, standard deviation.

### Supportive time-to-event and competing-risk analyses

**Figure 5:**
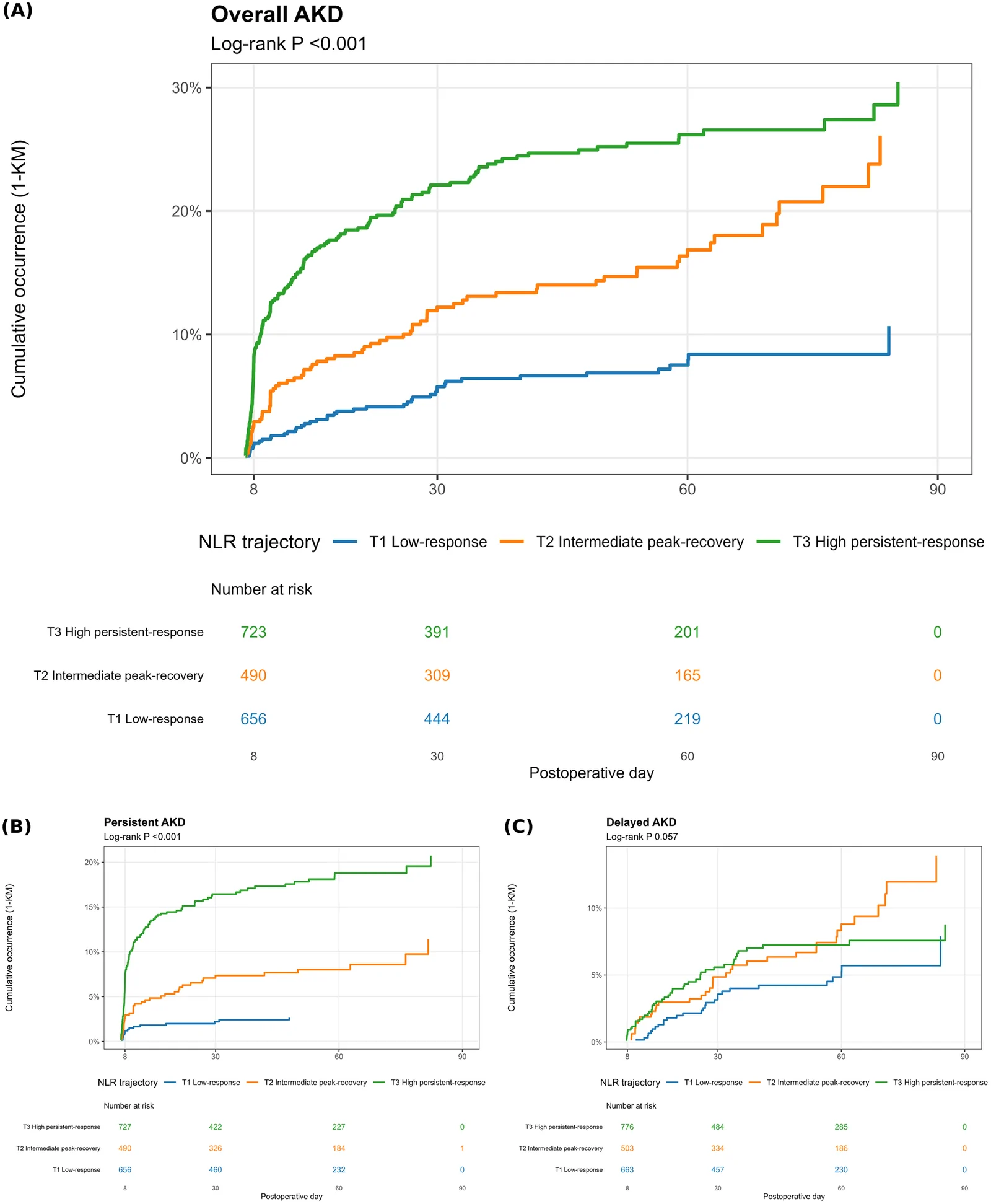
Kaplan–Meier cumulative-occurrence curves for overall, persistent, and delayed AKD. **(A)** Overall AKD. Curves show cumulative occurrence from postoperative day 8 through day 90 in the AKD-assessable cohort. Patients were censored at death, last available creatinine measurement, or day 90. The tables beneath the plots show numbers at risk. **(B)** Persistent AKD. Curves show cumulative occurrence of persistent AKD, defined as AKD during postoperative days 8–90 after early postoperative AKI, in the AKD-assessable cohort. Patients were censored at death, last available creatinine measurement, or day 90. The tables beneath the plots show numbers at risk. **(C)** Delayed AKD. Curves show cumulative occurrence of delayed AKD, defined as AKD during postoperative days 8–90 without early postoperative AKI, in the AKD-assessable cohort. Patients were censored at death, last available creatinine measurement, or day 90. The tables beneath the plots show numbers at risk. AKD, acute kidney disease; AKI, acute kidney injury; NLR, neutrophil-to-lymphocyte ratio.

Kaplan–Meier cumulative-occurrence curves differed across trajectories for overall AKD and persistent AKD (both log-rank *P* < 0.001), whereas the global comparison for delayed AKD was weaker (*P* = 0.057) (Figure 5). In adjusted Cox models, the HRs for overall AKD were 1.89 (95% CI: 1.29–2.78; *P* = 0.001) for T2 vs. T1 and 1.42 (95% CI: 1.07–1.90; *P* = 0.016) for T3 vs. T2. Corresponding HRs were 2.39 (95% CI: 1.32–4.32; *P* = 0.004) and 1.73 (95% CI: 1.19–2.53; *P* = 0.004) for persistent AKD, and 1.75 (95% CI: 1.05–2.91; *P* = 0.032) and 0.88 (95% CI: 0.55–1.40; *P* = 0.593) for delayed AKD (Table 5). In the exact-time Fine–Gray analysis, 46 deaths occurred before AKD; sHRs for overall AKD were 1.91 (95% CI: 1.29–2.81; *P* = 0.001) for T2 vs. T1 and 1.41 (95% CI: 1.06–1.87; *P* = 0.019) for T3 vs. T2 (Table 5).

**Table 5:** Time-to-event and competing-risk analyses for AKD outcomes.

Outcome	Model	Contrast	Estimate (95% CI)	*P* value	*n*/events
Overall AKD	Cox	T2 vs. T1	HR 1.89 (1.29–2.78)	0.001	1,975/298
Overall AKD	Cox	T3 vs. T2	HR 1.42 (1.07–1.90)	0.016	1,975/298
Persistent AKD	Cox	T2 vs. T1	HR 2.39 (1.32–4.32)	0.004	1,975/186
Persistent AKD	Cox	T3 vs. T2	HR 1.73 (1.19–2.53)	0.004	1,975/186
Delayed AKD	Cox	T2 vs. T1	HR 1.75 (1.05–2.91)	0.032	1,975/112
Delayed AKD	Cox	T3 vs. T2	HR 0.88 (0.55–1.40)	0.593	1,975/112
Overall AKD	Fine–Gray	T2 vs. T1	sHR 1.91 (1.29–2.81)	0.001	1,975/298
Overall AKD	Fine–Gray	T3 vs. T2	sHR 1.41 (1.06–1.87)	0.019	1,975/298

Cox and Fine–Gray models were adjusted for the primary covariate set and pooled across 20 imputed datasets. The Fine–Gray model used exact mortality time and included 46 deaths before AKD as competing events. AKD, acute kidney disease; CI, confidence interval; HR, hazard ratio; sHR, subdistribution hazard ratio.

### Sensitivity analyses

Complete-case and IPW analyses were consistent with the primary findings (Supplementary Tables S9, S10). When postoperative-day-0–7 AKI stage was added, associations of T2 and T3 vs. T1 with overall AKD remained, whereas the T3-vs.-T2 contrast was attenuated (OR: 1.29, 95% CI: 0.91–1.82; *P* = 0.146) (Supplementary Table S11). Expanded adjustment for intraoperative mean arterial pressure, vasoactive-drug use, and transfusion produced similar estimates (Supplementary Table S12). Measured perioperative and medication exposures are summarized in Supplementary Table S13. After additional adjustment for these exposures, T2 vs. T1 remained associated with overall AKD (OR: 1.96, 95% CI: 1.30–2.95), while T3 vs. T2 was attenuated (OR: 1.37, 95% CI: 0.98–1.90) but remained associated with persistent AKD (OR: 1.73, 95% CI: 1.15–2.62) (Supplementary Table S14).

### Exploratory outcomes

Mortality increased across trajectories in the full cohort. Ninety-day mortality was 1.0% (8/799), 2.2% (13/588), and 8.4% (74/878) in T1, T2, and T3, respectively. In parsimoniously adjusted bias-reduced models, T3 was associated with higher 90-day mortality than T1 (OR: 5.68, 95% CI: 2.72–11.85) and T2 (OR: 3.49, 95% CI: 1.90–6.40), whereas T2 vs. T1 was not statistically significant (OR: 1.63, 95% CI: 0.69–3.86). Postoperative in-hospital hemodialysis/CRRT records within 90 days occurred in 2.4%, 7.1%, and 15.5%; adjusted ORs were 2.36 (95% CI: 1.10–5.08) for T2 vs. T1 and 3.67 (95% CI: 2.17–6.19) for T3 vs. T2 (Table 6; Supplementary Tables S15A,B and S16). These rare outcomes were exploratory.

**Table 6:** Exploratory patient-centered outcomes.

Outcome	Contrast	Adjusted OR (95% CI)	*P* value	*n*	Events
90-day mortality	T2 vs. T1	OR 1.63 (0.69–3.86)	0.270	2,265	95
90-day mortality	T3 vs. T1	OR 5.68 (2.72–11.85)	<0.001	2,265	95
90-day mortality	T3 vs. T2	OR 3.49 (1.90–6.40)	<0.001	2,265	95
In-hospital HD/CRRT record	T2 vs. T1	OR 2.36 (1.10–5.08)	0.028	2,265	197
In-hospital HD/CRRT record	T3 vs. T1	OR 8.66 (4.35–17.23)	<0.001	2,265	197
In-hospital HD/CRRT record	T3 vs. T2	OR 3.67 (2.17–6.19)	<0.001	2,265	197

Bias-reduced logistic regression used a parsimonious adjustment set (age, sex, ASA physical status ≥3, emergency surgery, baseline creatinine, and surgery type). The HD/CRRT endpoint denotes positive postoperative inpatient ward records within 90 days and is not complete post-discharge 90-day renal-replacement-therapy surveillance. ASA, American Society of Anesthesiologists; CI, confidence interval; CRRT, continuous renal replacement therapy; HD, hemodialysis; OR, odds ratio.

The three-class solution showed high internal reproducibility in repeated five-fold cross-validation. All 25 refitted models converged, with 89.8% pooled held-out agreement with the full-cohort classification (Cohen's kappa = 0.847). Class-specific agreement was 90.7%, 92.7%, and 87.1% for T1, T2, and T3, respectively. Trajectory shapes were highly consistent across refits, and 86.9% of patients received the same class in all five repeats (Supplementary Table S17).

In an additional *post-hoc* exploratory analysis, late postoperative NLR showed moderate discrimination for overall AKD (AUC 0.711; bootstrap 95% CI, 0.680–0.743), with a bootstrap median threshold of 7.91 (95% interval, 5.89–9.18). Discrimination was weaker for persistent AKD among patients with early AKI and intermediate for delayed AKD among patients without early AKI (Supplementary Table S18).

## Discussion

In this single-center cohort, three data-driven perioperative NLR patterns were associated with renal dysfunction beyond the conventional 7-day AKI window. T1 represented the lowest inflammatory burden and the lowest AKD occurrence. T2 combined a higher preoperative level with a pronounced postoperative peak that subsequently declined, yet it remained associated with overall, persistent, and delayed AKD relative to T1. T3 combined higher baseline inflammation with sustained postoperative elevation and showed the clearest graded association with persistent AKD. These observations identify sustained NLR elevation as a marker of impaired renal recovery after cardiac surgery. The non-monotonic pattern for delayed AKD, together with attenuation after early-AKI and medication adjustment, argues against a simple causal or dose-response interpretation. The trajectories should therefore be viewed as cohort-specific phenotypes and potential risk markers rather than validated clinical classes.

NLR is an integrated but nonspecific measure of the perioperative immune response (8, 10). Cardiopulmonary bypass, ischemia–reperfusion injury, surgical tissue damage, and exposure of blood to nonphysiological surfaces can initiate sterile inflammatory signaling, including the release of damage-associated molecular patterns and cytokine-mediated leukocyte activation (6, 29). The window-specific profiles help distinguish baseline susceptibility from the postoperative response: median preoperative NLR was 1.64, 2.31, and 3.46 in T1, T2, and T3, whereas median NLR at 72–168 h was 3.57, 6.77, and 11.71. Thus, both pre-existing inflammation and failure of postoperative resolution may contribute. Postoperative neutrophilia may reflect the recruitment of mature neutrophils through demargination, whereas lymphopenia may reflect reduced lymphocyte numbers and increased T-cell apoptosis after cardiopulmonary bypass (30, 31). An elevated NLR can therefore result from several distinct processes and should not be interpreted as a direct measure of a single inflammatory pathway (8, 10, 30, 31). Nevertheless, neutrophil activation provides a plausible link with kidney injury through enhanced reactive oxygen species production, adhesion-dependent recruitment, and neutrophil extracellular trap (NET) formation (30). A recent translational study detected CD11b expression and NET formation in patients with AKI after cardiac surgery and showed in renal ischemia–reperfusion models that genetic deletion or antibody blockade of Mac-1 reduced NET formation and kidney injury (32). NLR trajectories may therefore capture clinically relevant differences in the intensity and persistence of perioperative immune stress.

The stronger association with persistent AKD is biologically plausible because recovery from AKI is an active process rather than a passive return of serum creatinine toward baseline (33, 34). Successful recovery requires resolution of inflammation and repair and redifferentiation of injured tubular epithelial cells. When inflammatory signaling persists, tubular cells may remain in maladaptive injury states, endothelial dysfunction may impair oxygen delivery, and continued monocyte and macrophage recruitment may favor fibroblast activation and interstitial remodeling (35–37). The T3 pattern may capture this sustained systemic stress. Human studies after cardiac surgery support components of this model. Lower urinary epidermal growth factor, a marker of tubular health, and higher monocyte chemoattractant protein-1, a marker of intrarenal inflammation, have been associated with subsequent kidney function loss (34). More broadly, longitudinal biomarker studies of AKI have linked persistent tubular injury and inflammation, together with slower restoration of tubular health, to kidney disease progression (36). Experimental single-cell and epigenomic studies also indicate that successful and failed repair follow distinct cellular programs after AKI (35, 37). Preoperative vascular dysfunction may further lower the kidney's capacity to tolerate perioperative hypoperfusion and inflammatory stress (38). This framework offers a plausible explanation for the particularly strong association between the persistently high NLR trajectory and persistent AKD. However, NLR trajectories and early AKI were both defined within postoperative days 0–7. Early kidney injury can itself alter neutrophil and lymphocyte counts, while surgical severity and hemodynamic instability can cause both phenomena. The attenuation of some adjacent contrasts after adding early AKI stage supports temporal interdependence, not an AKI-independent or causal NLR effect.

T2 remained associated with delayed AKD vs. T1 despite subsequent NLR decline, whereas T3 did not show a further increase over T2. A transient but marked inflammatory response may signal perioperative tissue injury, hemodynamic stress, or treatment intensity that leaves residual renal vulnerability after creatinine-defined early AKI has resolved or was never detected. Delayed AKD is also heterogeneous: some cases may reflect subclinical perioperative injury, while others may result from later infection, heart failure, altered volume status, nephrotoxic exposure, or readmission-related illness (3, 23, 39). These later determinants were incompletely measured, and only 112 delayed events occurred. The observed T2–T3 plateau may therefore reflect etiologic heterogeneity, residual confounding, imprecision, or a genuinely non-linear relationship; it should not be interpreted as evidence that more sustained inflammation protects against delayed AKD.

The summary measures complement the trajectory analysis by separating, to some extent, the overall NLR level from within-patient variation. Mean perioperative NLR and late postoperative NLR showed the most consistent associations with AKD, whereas the associations with NLR SD were smaller and NLR CV was not associated with the outcomes. The concordance between the trajectory and summary analyses suggests that the absolute magnitude and persistence of NLR elevation were more informative than proportional fluctuation. Because SD and CV are influenced by the underlying NLR level and sampling frequency, these findings indicate that absolute magnitude and persistence were more informative in this dataset, rather than that short-term variability is biologically irrelevant.

Modeling AKD in several ways addressed different aspects of outcome ascertainment. Logistic models evaluated occurrence during the prespecified period, Cox models incorporated the timing of the qualifying measurement, and the exact-time Fine–Gray model treated death before AKD as a competing event (28). Kaplan–Meier curves visually demonstrated the separation for overall and persistent AKD. Concordant estimates across these approaches make it less likely that the associations depended on one outcome formulation.

The exploratory mortality and inpatient hemodialysis/CRRT findings provide patient-centered context but should be interpreted cautiously. The concentration of these outcomes in T3 is consistent with a trajectory that marks greater perioperative illness burden; it does not establish that sustained NLR elevation causes death or dialysis. Event counts were limited, adjusted models were intentionally parsimonious, and the dialysis source was restricted to inpatient ward documentation. The exploratory threshold analysis showed that a single late NLR value provided only moderate discrimination, with lower performance for persistent AKD and differing thresholds across AKD phenotypes. These findings support late NLR as a potential risk-enrichment marker but argue against reducing the longitudinal trajectories to a single universal cutoff.

NLR is inexpensive, routinely available, and readily repeatable, but its potential clinical role would be risk enrichment rather than standalone prediction (8, 40). If externally validated, persistently elevated perioperative NLR could help identify patients for whom structured serum creatinine surveillance during postoperative days 8–90, closer review of volume status and medications, or earlier follow-up may be warranted (39, 41). The present study does not show that NLR-guided monitoring or anti-inflammatory treatment improves renal outcomes, and the exploratory thresholds identified here should not be interpreted as validated clinical cutoffs. The most informative next step would be prospective validation with prespecified blood and creatinine sampling. Combining NLR trajectories with markers of tubular injury and repair, such as urinary epidermal growth factor, monocyte chemoattractant protein-1, and other tubular stress markers, as well as measurements of cytokine activity, neutrophil activation, and lymphocyte subsets, may help distinguish a failure of immune resolution from nonspecific postoperative stress or intercurrent complications (30–32).

This study has several limitations. First, the retrospective, single-center design precludes causal inference. Although the three-class solution showed high internal reproducibility in repeated cross-validation, the trajectories remain data-driven and require external validation before clinical implementation. Second, despite expanded sensitivity analyses, residual confounding remains. Available data permitted adjustment for intraoperative mean arterial pressure, vasoactive drugs, transfusion, systemic corticosteroids, selected inpatient preoperative medications, and candidate nephrotoxins, but infection, outpatient medication history, exact dose and timing, and several postoperative complications were unavailable. Postoperative exposures may also be mediators or markers of illness severity. Third, NLR and early AKI were defined over the same first postoperative week; reverse influence of kidney injury on leukocyte counts cannot be excluded. Fourth, AKD ascertainment relied on clinically obtained creatinine measurements. Included and excluded patients differed substantially, and IPW addresses only measured determinants of surveillance. Although 98.7% of AKD cases had at least two creatinine measurements and 71.5% had at least two qualifying values, 28.5% were supported by one qualifying value; irregular testing still limits inference about onset and duration. Fifth, maximum-posterior-probability class assignment did not propagate classification uncertainty into outcome models, particularly relevant for T2. Sixth, mortality and inpatient hemodialysis/CRRT analyses were exploratory, involved uncommon endpoints, and the ward-recorded dialysis variable did not capture complete post-discharge 90-day renal-replacement therapy. Finally, urine output and direct markers of neutrophil activation, intrarenal inflammation, and tubular repair were unavailable.

## Conclusion

In adults undergoing cardiac surgery, perioperative NLR trajectories were associated with creatinine-defined AKD during postoperative days 8–90, with the clearest graded relationship observed for persistent AKD. Concordant associations for mean perioperative and late postoperative NLR indicate that the magnitude and persistence of NLR elevation were more informative than relative within-patient variability. Sustained NLR elevation may be a candidate marker of impaired renal recovery, but its clinical utility for directing postoperative kidney surveillance requires prospective validation. Prospective multicenter studies incorporating standardized creatinine follow-up and direct immune and renal injury biomarkers should evaluate its clinical utility and clarify the pathways linking persistent perioperative immune stress to impaired renal recovery.

## Data Availability

The datasets presented in this study can be found in online repositories. The names of the repository/repositories and accession number(s) can be found below: https://physionet.org/content/inspire/1.4.2/.
