# Supplementary material for: Perioperative neutrophil-to-lymphocyte ratio trajectories and creatinine-defined acute kidney disease after cardiac surgery: a study using the INSPIRE database
Source: Front Cardiovasc Med. 2026 Sep 8;13:1952199. doi: 10.3389/fcvm.2026.1952199 (PMC13593457; doi:10.3389/fcvm.2026.1952199)
Supplement: Supplementary file 1 [file Datasheet1.docx]

Supplementary Material

**Supplementary Table 1. NLR window coverage.**

| **Metric** | **n** | **% of primary cohort** |
| --- | --- | --- |
| Primary cardiac surgery cohort | 2346 | 100.0 |
| Preoperative NLR available | 2080 | 88.7 |
| Postoperative 0–24 h NLR available | 2336 | 99.6 |
| Postoperative 24–48 h NLR available | 2292 | 97.7 |
| Postoperative 48–72 h NLR available | 2210 | 94.2 |
| Postoperative 72–168 h NLR available | 2239 | 95.4 |
| Eligible for trajectory modeling (≥4 of 5 NLR windows) | 2265 | 96.5 |
| Complete NLR data in all 5 windows | 1884 | 80.3 |

The preoperative NLR window was defined as the latest available measurement within 7 days before surgery. NLR values were not imputed for trajectory modeling; patients were required to have observed NLR values in at least four of the five predefined windows. NLR, neutrophil-to-lymphocyte ratio.

**Supplementary Table 2. GBTM model selection.**

| **Model** | **BIC** | **AIC** | **Minimum class %** | **Minimum AvePP** | **Valid model** |
| --- | --- | --- | --- | --- | --- |
| 5 classes, quadratic | 27506.1 | 27330.9 | 15.4 | 0.610 | No |
| 6 classes, quadratic | 27549.5 | 27337.8 | 7.0 | 0.471 | No |
| 4 classes, quadratic | 27711.4 | 27572.7 | 15.7 | 0.619 | No |
| 3 classes, quadratic | 27856.3 | 27754.1 | 26.0 | 0.731 | Yes; selected |
| 2 classes, quadratic | 28287.9 | 28222.2 | 47.9 | 0.927 | Yes |
| 1 class, quadratic | 30856.9 | 30827.7 | 100.0 | 1.000 | No |
| 3 classes, linear | 34127.1 | 34054.1 | 2.6 | 0.823 | No |
| 4 classes, linear | 34162.7 | 34060.5 | 2.1 | 0.506 | No |
| 5 classes, linear | 34196.4 | 34065.0 | 2.6 | 0.335 | No |
| 6 classes, linear | 34226.9 | 34066.2 | 3.1 | 0.275 | No |
| 2 classes, linear | 34238.2 | 34187.1 | 35.2 | 0.847 | Yes |
| 1 class, linear | 35054.9 | 35033.0 | 100.0 | 1.000 | No |

GBTM, group-based trajectory modeling; BIC, Bayesian information criterion; AIC, Akaike information criterion; AvePP, average posterior probability of class membership. Candidate models used log2-transformed perioperative NLR values. The final model was selected according to overall model fit, minimum class size, classification quality, separation of trajectory patterns, and clinical interpretability. The 3-class quadratic model was selected for subsequent analyses.

**Supplementary Table 3. Selected characteristics of patients with and without postoperative day-8–90 creatinine follow-up.**

| **Characteristic** | **Included (n=1,975)** | **Excluded (n=290)** | **SMD** | **P value** |
| --- | --- | --- | --- | --- |
| Age, years | 1975; 65.00 [55.00, 75.00] | 290; 55.00 [40.00, 65.00] | 0.620 | <0.001 |
| BMI, kg/m² | 1971; 23.44 [20.81, 25.71] | 290; 23.44 [21.48, 25.95] | -0.124 | 0.066 |
| Baseline creatinine, mg/dL | 1975; 0.88 [0.74, 1.15] | 290; 0.80 [0.71, 0.98] | 0.330 | <0.001 |
| CPB duration, min | 1845; 200.00 [155.00, 245.00] | 280; 175.00 [138.75, 215.00] | 0.347 | <0.001 |
| Preoperative NLR | 1765; 2.31 [1.64, 3.56] | 257; 1.87 [1.31, 2.48] | 0.317 | <0.001 |
| NLR at 72–168 h | 1941; 6.42 [4.07, 11.71] | 270; 4.31 [3.04, 6.37] | 0.556 | <0.001 |
| Female sex | 742/1975 (37.6%) | 132/290 (45.5%) | -0.162 | 0.011 |
| Early postoperative AKI | 580/1975 (29.4%) | 63/290 (21.7%) | 0.176 | 0.009 |
| CABG | 765/1975 (38.7%) | 9/290 (3.1%) | 0.974 | <0.001 |
| Valve surgery | 1060/1975 (53.7%) | 249/290 (85.9%) | -0.748 | <0.001 |

*Values are n/N (%) or n; median [IQR], as appropriate. P values are descriptive. SMD, standardized mean difference; AKI, acute kidney injury; BMI, body mass index; CABG, coronary artery bypass grafting; CPB, cardiopulmonary bypass; NLR, neutrophil-to-lymphocyte ratio.*

**Supplementary Table 4. Creatinine-measurement support among AKD cases.**

| **Metric** | **Value** |
| --- | --- |
| AKD cases | 298 |
| Day-8–90 creatinine measurements, median [IQR] | 17 [7–39] |
| Cases with ≥2 creatinine measurements | 294/298 (98.7%) |
| Cases with ≥2 values independently meeting AKD criteria | 213/298 (71.5%) |
| Cases identified by one qualifying value | 85/298 (28.5%) |

*Counts refer to clinically obtained serum creatinine values during postoperative days 8–90. AKD, acute kidney disease;* *IQR, interquartile range.*

**Supplementary Table 5. Posterior-probability diagnostics and class distribution for the selected three-class model.**

| **Trajectory** | **n** | **AvePP (mean max PP)** | **Median [IQR] max PP** | **Minimum max PP** | **Max PP ≥0.70** | **Max PP ≥0.90** | **Mean normalized entropy** |
| --- | --- | --- | --- | --- | --- | --- | --- |
| T1 | 799 | 0.880 | 0.963 [0.799, 0.997] | 0.386 | 663 (83.0%) | 512 (64.1%) | 0.268 |
| T2 | 588 | 0.731 | 0.742 [0.640, 0.839] | 0.400 | 370 (62.9%) | 35 (6.0%) | 0.576 |
| T3 | 878 | 0.895 | 0.983 [0.822, 1.000] | 0.370 | 751 (85.5%) | 589 (67.1%) | 0.220 |

*Trajectory names were low-response (T1), intermediate peak-recovery (T2), and high persistent-response (T3). AvePP values correspond to mean maximum posterior probabilities within assigned classes. Patients were assigned by maximum posterior probability. Lower maximum posterior probability and higher entropy indicate greater classification uncertainty. AvePP, average posterior probability; IQR, interquartile range; PP, posterior probability.*

**Supplementary Table 6. Window-specific NLR distributions by trajectory.**

| **Trajectory** | **Window** | **Available n** | **Median [IQR] NLR** |
| --- | --- | --- | --- |
| T1 | Preoperative | 726 | 1.64 [1.31, 2.22] |
| T1 | 0–24 h | 797 | 5.33 [4.14, 8.12] |
| T1 | 24–48 h | 791 | 7.91 [6.25, 9.98] |
| T1 | 48–72 h | 745 | 5.42 [4.19, 6.77] |
| T1 | 72–168 h | 758 | 3.57 [2.76, 4.56] |
| T2 | Preoperative | 578 | 2.31 [1.79, 3.05] |
| T2 | 0–24 h | 587 | 8.59 [6.21, 12.03] |
| T2 | 24–48 h | 588 | 12.80 [11.41, 16.59] |
| T2 | 48–72 h | 587 | 11.11 [8.34, 12.40] |
| T2 | 72–168 h | 583 | 6.77 [5.27, 8.94] |
| T3 | Preoperative | 718 | 3.46 [2.19, 6.41] |
| T3 | 0–24 h | 878 | 16.07 [9.74, 26.35] |
| T3 | 24–48 h | 875 | 16.59 [12.40, 26.35] |
| T3 | 48–72 h | 863 | 16.07 [9.98, 26.35] |
| T3 | 72–168 h | 870 | 11.71 [6.59, 16.59] |

*These descriptive distributions aid interpretation of the longitudinal classes and are not validated cutoffs for individual assignment. IQR, interquartile range; NLR, neutrophil-to-lymphocyte ratio.*

**Supplementary Table 7. Diagnostics of inverse probability weights for AKD ascertainment.**

| **Metric** | **Value** |
| --- | --- |
| NLR-eligible trajectory cohort | 2,265 |
| AKD-assessable cohort | 1,975 |
| Without AKD ascertainment | 290 |
| Marginal probability of AKD ascertainment | 0.872 |
| Minimum predicted probability of AKD ascertainment | 0.072 |
| Maximum predicted probability of AKD ascertainment | 0.990 |
| Minimum truncated stabilized weight | 0.881 |
| 25th percentile of weight | 0.881 |
| Median weight | 0.907 |
| 75th percentile of weight | 1.054 |
| Maximum truncated stabilized weight | 1.712 |
| Unweighted effective sample size | 1,975 |
| Raw IPW effective sample size | 1911.5 |
| Truncated IPW effective sample size | 1916.9 |

Stabilized inverse probability weights were calculated as the marginal probability of AKD ascertainment divided by the individual predicted probability of AKD ascertainment and were truncated at the 1st and 99th percentiles. Weights were estimated in the NLR-eligible trajectory cohort and applied to patients with ascertainable AKD status. AKD, acute kidney disease; IPW, inverse probability weighting; NLR, neutrophil-to-lymphocyte ratio.

**Supplementary Table 8. Inverse probability-weighted sensitivity analysis accounting for AKD ascertainment.**

| **Outcome** | **Contrast** | **n in model** | **Events** | **IPW-adjusted OR (95% CI)** | **P value** |
| --- | --- | --- | --- | --- | --- |
| Overall AKD | T2 vs T1 | 1187 | 120 | 2.10 (1.36–3.24) | <0.001 |
| Overall AKD | T3 vs T2 | 1300 | 254 | 1.43 (1.03–1.99) | 0.032 |
| Stage 2–3 AKD | T2 vs T1 | 1187 | 43 | 2.94 (1.42–6.07) | 0.004 |
| Stage 2–3 AKD | T3 vs T2 | 1300 | 118 | 1.44 (0.92–2.27) | 0.114 |
| Persistent AKD | T2 vs T1 | 1187 | 55 | 2.75 (1.42–5.34) | 0.003 |
| Persistent AKD | T3 vs T2 | 1300 | 170 | 1.80 (1.19–2.73) | 0.005 |
| Delayed AKD | T2 vs T1 | 1187 | 65 | 1.61 (0.91–2.84) | 0.101 |
| Delayed AKD | T3 vs T2 | 1300 | 84 | 0.92 (0.55–1.52) | 0.737 |

Weighted logistic regression models were fitted among patients with ascertainable AKD status using stabilized inverse probability weights for AKD ascertainment. The same covariate adjustment set as the primary analysis was used. The n in model reflects the number of patients included in each adjacent trajectory contrast. AKD, acute kidney disease; CI, confidence interval; IPW, inverse probability weighting; OR, odds ratio; T1, low-response trajectory; T2, intermediate peak-recovery trajectory; T3, high persistent-response trajectory.

**Supplementary Table 9. Complete-case sensitivity analysis for main AKD outcomes.**

| **Outcome** | **Contrast** | **OR (95% CI)** | **P value** | **n** | **Events** |
| --- | --- | --- | --- | --- | --- |
| Overall AKD | T2 vs T1 | 1.96 (1.28-3.03) | 0.002 | 1750 | 264 |
| Overall AKD | T3 vs T2 | 1.49 (1.06-2.10) | 0.023 | 1750 | 264 |
| Stage 2-3 AKD | T2 vs T1 | 3.28 (1.51-7.95) | 0.005 | 1750 | 110 |
| Stage 2-3 AKD | T3 vs T2 | 1.51 (0.92-2.54) | 0.110 | 1750 | 110 |

Complete-case analyses were performed among patients with complete data for all covariates in the fully adjusted model. Models were adjusted for age, sex, BMI, ASA physical status, emergency surgery, baseline creatinine, hypertension, diabetes, heart failure, myocardial infarction, surgery type, operation duration, and CPB duration. OR, odds ratio; AKD, acute kidney disease.

**Supplementary Table 10. Complete-case sensitivity analysis for AKD subtypes.**

| **Outcome** | **Contrast** | **OR (95% CI)** | **P value** | **n** |
| --- | --- | --- | --- | --- |
| Persistent AKD | T2 vs T1 | 2.78 (1.39-5.53) | 0.004 | 1750 |
| Delayed AKD | T2 vs T1 | 1.69 (0.98-2.91) | 0.059 | 1750 |
| Persistent AKD | T3 vs T2 | 1.88 (1.21-2.91) | 0.005 | 1750 |
| Delayed AKD | T3 vs T2 | 1.01 (0.61-1.68) | 0.968 | 1750 |

Complete-case multinomial logistic regression was performed with no AKD as the reference outcome category. Models were adjusted for the same covariates as the primary fully adjusted model. OR, odds ratio; AKD, acute kidney disease.

**Supplementary Table 11. Sensitivity analysis additionally adjusted for postoperative day-0–7 AKI stage.**

| **Outcome** | **Contrast** | **Adjusted OR (95% CI)** | **P value** | **n** | **Events** |
| --- | --- | --- | --- | --- | --- |
| Overall AKD | T2 vs T1 | 1.83 (1.20-2.80) | 0.005 | 1975 | 298 |
| Overall AKD | T3 vs T1 | 2.36 (1.55-3.58) | <0.001 | 1975 | 298 |
| Overall AKD | T3 vs T2 | 1.29 (0.91-1.82) | 0.146 | 1975 | 298 |
| Stage 2–3 AKD | T2 vs T1 | 2.33 (1.12-4.88) | 0.024 | 1975 | 130 |
| Stage 2–3 AKD | T3 vs T1 | 2.97 (1.45-6.10) | 0.003 | 1975 | 130 |
| Stage 2–3 AKD | T3 vs T2 | 1.27 (0.76-2.13) | 0.359 | 1975 | 130 |
| Persistent AKD | T2 vs T1 | 1.94 (0.93-4.02) | 0.076 | 1975 | 186 |
| Persistent AKD | T3 vs T1 | 3.00 (1.50-6.01) | 0.002 | 1975 | 186 |
| Persistent AKD | T3 vs T2 | 1.55 (0.93-2.59) | 0.096 | 1975 | 186 |
| Delayed AKD | T2 vs T1 | 1.83 (1.07-3.12) | 0.027 | 1975 | 112 |
| Delayed AKD | T3 vs T1 | 2.01 (1.15-3.51) | 0.014 | 1975 | 112 |
| Delayed AKD | T3 vs T2 | 1.10 (0.67-1.81) | 0.707 | 1975 | 112 |

*The primary covariate set was supplemented with early postoperative AKI stage. Because early AKI temporally overlaps NLR trajectory definition and may be a mediator or consequence of shared illness severity, these estimates are not interpreted as causal mediation effects.*

**Supplementary Table 12. Sensitivity analysis additionally adjusted for measured hemodynamic and transfusion variables.**

| **Outcome** | **Contrast** | **Adjusted OR (95% CI)** | **P value** | **n** | **Events** |
| --- | --- | --- | --- | --- | --- |
| Overall AKD | T2 vs T1 | 1.99 (1.33-3.00) | <0.001 | 1975 | 298 |
| Overall AKD | T3 vs T1 | 2.85 (1.92-4.25) | <0.001 | 1975 | 298 |
| Overall AKD | T3 vs T2 | 1.43 (1.04-1.98) | 0.030 | 1975 | 298 |
| Stage 2–3 AKD | T2 vs T1 | 2.69 (1.34-5.41) | 0.005 | 1975 | 130 |
| Stage 2–3 AKD | T3 vs T1 | 3.88 (1.97-7.65) | <0.001 | 1975 | 130 |
| Stage 2–3 AKD | T3 vs T2 | 1.44 (0.90-2.30) | 0.124 | 1975 | 130 |
| Persistent AKD | T2 vs T1 | 2.42 (1.31-4.45) | 0.005 | 1975 | 186 |
| Persistent AKD | T3 vs T1 | 4.30 (2.41-7.67) | <0.001 | 1975 | 186 |
| Persistent AKD | T3 vs T2 | 1.78 (1.19-2.67) | 0.005 | 1975 | 186 |
| Delayed AKD | T2 vs T1 | 1.75 (1.03-2.96) | 0.039 | 1975 | 112 |
| Delayed AKD | T3 vs T1 | 1.57 (0.89-2.75) | 0.116 | 1975 | 112 |
| Delayed AKD | T3 vs T2 | 0.90 (0.55-1.46) | 0.667 | 1975 | 112 |

*The expanded model added mean intraoperative arterial pressure, any intraoperative vasoactive-drug use, and any perioperative transfusion to the primary covariate set.*

**Supplementary Table 13. Measured perioperative and medication exposures by NLR trajectory.**

| **Variable** | **T1 (n=799)** | **T2 (n=588)** | **T3 (n=878)** |
| --- | --- | --- | --- |
| Mean intraoperative MAP, median [IQR], mmHg | 70.8 [67.2, 74.5] | 69.9 [66.6, 73.4] | 67.8 [64.3, 71.9] |
| Any intraoperative vasoactive drug | 24/799 (3.0%) | 19/588 (3.2%) | 58/878 (6.6%) |
| Any perioperative transfusion | 514/799 (64.3%) | 444/588 (75.5%) | 679/878 (77.3%) |
| Preoperative RAAS inhibitor within 7 d | 178/799 (22.3%) | 165/588 (28.1%) | 184/878 (21.0%) |
| Preoperative diuretic within 7 d | 183/799 (22.9%) | 216/588 (36.7%) | 472/878 (53.8%) |
| Postoperative vasoactive drug, days 0–7 | 272/799 (34.0%) | 250/588 (42.5%) | 424/878 (48.3%) |
| Systemic corticosteroid, day −1 to day 7 | 79/799 (9.9%) | 65/588 (11.1%) | 226/878 (25.7%) |
| Candidate nephrotoxin, days 0–7 | 457/799 (57.2%) | 369/588 (62.8%) | 707/878 (80.5%) |

*Medication variables are inpatient records within the stated windows and do not represent complete outpatient medication history. Candidate nephrotoxins included selected aminoglycosides, glycopeptides, amphotericin, and nonsteroidal anti-inflammatory agents identified from available medication text. IQR, interquartile range; MAP, mean arterial pressure; NLR, neutrophil-to-lymphocyte ratio; RAAS, renin–angiotensin–aldosterone system.*

**Supplementary Table 14. Sensitivity analysis additionally adjusted for measured perioperative and medication exposures.**

| **Outcome** | **Contrast** | **Adjusted OR (95% CI)** | **P value** | **n** | **Events** |
| --- | --- | --- | --- | --- | --- |
| Overall AKD | T2 vs T1 | 1.96 (1.30-2.95) | 0.001 | 1975 | 298 |
| Overall AKD | T3 vs T1 | 2.68 (1.79-4.01) | <0.001 | 1975 | 298 |
| Overall AKD | T3 vs T2 | 1.37 (0.98-1.90) | 0.062 | 1975 | 298 |
| Stage 2–3 AKD | T2 vs T1 | 2.50 (1.24-5.04) | 0.010 | 1975 | 130 |
| Stage 2–3 AKD | T3 vs T1 | 3.38 (1.70-6.70) | <0.001 | 1975 | 130 |
| Stage 2–3 AKD | T3 vs T2 | 1.35 (0.84-2.18) | 0.217 | 1975 | 130 |
| Persistent AKD | T2 vs T1 | 2.38 (1.29-4.39) | 0.005 | 1975 | 186 |
| Persistent AKD | T3 vs T1 | 4.12 (2.30-7.39) | <0.001 | 1975 | 186 |
| Persistent AKD | T3 vs T2 | 1.73 (1.15-2.62) | 0.009 | 1975 | 186 |
| Delayed AKD | T2 vs T1 | 1.72 (1.01-2.93) | 0.046 | 1975 | 112 |
| Delayed AKD | T3 vs T1 | 1.43 (0.81-2.55) | 0.217 | 1975 | 112 |
| Delayed AKD | T3 vs T2 | 0.83 (0.51-1.37) | 0.477 | 1975 | 112 |

*The model added mean intraoperative arterial pressure, intraoperative vasoactive use, transfusion, inpatient preoperative RAAS-inhibitor and diuretic records, postoperative vasoactive and candidate-nephrotoxin records, and perioperative systemic corticosteroid records. Postoperative exposures may be mediators or markers of illness severity.*

**Supplementary Table 15A. Observed mortality by NLR trajectory.**

| **Outcome** | **T1 (n=799)** | **T2 (n=588)** | **T3 (n=878)** |
| --- | --- | --- | --- |
| 28-day all-cause mortality | 5/799 (0.6%) | 5/588 (0.9%) | 38/878 (4.3%) |
| 30-day all-cause mortality | 5/799 (0.6%) | 5/588 (0.9%) | 43/878 (4.9%) |
| 90-day all-cause mortality | 8/799 (1.0%) | 13/588 (2.2%) | 74/878 (8.4%) |
| In-hospital mortality | 7/799 (0.9%) | 19/588 (3.2%) | 86/878 (9.8%) |

*Values are deaths/N (%). NLR, neutrophil-to-lymphocyte ratio.*

**Supplementary Table 15B. Bias-reduced logistic regression for mortality outcomes.**

| **Outcome** | **Contrast** | **Adjusted OR (95% CI)** | **P value** | **n** | **Events** |
| --- | --- | --- | --- | --- | --- |
| 28-day all-cause mortality | T2 vs T1 | 1.00 (0.31-3.19) | 0.999 | 2265 | 48 |
| 28-day all-cause mortality | T3 vs T1 | 3.87 (1.55-9.67) | 0.004 | 2265 | 48 |
| 28-day all-cause mortality | T3 vs T2 | 3.87 (1.58-9.47) | 0.003 | 2265 | 48 |
| 30-day all-cause mortality | T2 vs T1 | 1.01 (0.32-3.23) | 0.987 | 2265 | 53 |
| 30-day all-cause mortality | T3 vs T1 | 4.40 (1.78-10.88) | 0.001 | 2265 | 53 |
| 30-day all-cause mortality | T3 vs T2 | 4.36 (1.80-10.57) | 0.001 | 2265 | 53 |
| 90-day all-cause mortality | T2 vs T1 | 1.63 (0.69-3.86) | 0.270 | 2265 | 95 |
| 90-day all-cause mortality | T3 vs T1 | 5.68 (2.72-11.85) | <0.001 | 2265 | 95 |
| 90-day all-cause mortality | T3 vs T2 | 3.49 (1.90-6.40) | <0.001 | 2265 | 95 |
| In-hospital mortality | T2 vs T1 | 2.88 (1.24-6.71) | 0.014 | 2265 | 112 |
| In-hospital mortality | T3 vs T1 | 7.84 (3.65-16.84) | <0.001 | 2265 | 112 |
| In-hospital mortality | T3 vs T2 | 2.72 (1.62-4.56) | <0.001 | 2265 | 112 |

*Models used a parsimonious adjustment set: age, sex, ASA physical status ≥3, emergency surgery, baseline creatinine, and surgery type. These rare-event analyses are exploratory.*

**Supplementary Table 16. Observed postoperative in-hospital hemodialysis/CRRT records by trajectory.**

| **Recorded interval** | **T1 (n=799)** | **T2 (n=588)** | **T3 (n=878)** |
| --- | --- | --- | --- |
| Recorded within postoperative days 0–90 | 19/799 (2.4%) | 42/588 (7.1%) | 136/878 (15.5%) |
| Recorded within days 0–7 | 18/799 (2.3%) | 41/588 (7.0%) | 120/878 (13.7%) |
| Recorded within days 8–90 | 2/799 (0.3%) | 7/588 (1.2%) | 64/878 (7.3%) |

*Positive records came from the inpatient ward-vitals source. These data do not provide complete post-discharge 90-day renal-replacement-therapy surveillance. CRRT, continuous renal replacement therapy.*

**Supplementary Table 17. Repeated five-fold internal cross-validation of the selected three-class quadratic GBTM.**

**A. Class-specific held-out reproducibility**

| Trajectory | Full-cohort n (%) | Held-out class agreement (%) | Mean held-out max PP | Max PP ≥0.70 (%) | Max PP ≥0.90 (%) | Mean shape correlation | Mean shape RMSE |
| --- | --- | --- | --- | --- | --- | --- | --- |
| T1 | 799 (35.3%) | 90.7 | 0.880 | 83.9 | 62.8 | 0.99987 | 0.037 |
| T2 | 588 (26.0%) | 92.7 | 0.731 | 61.1 | 7.0 | 0.99984 | 0.050 |
| T3 | 878 (38.8%) | 87.1 | 0.886 | 83.7 | 65.0 | 0.99985 | 0.039 |

**B. Overall and subject-level reproducibility**

| Metric | Value |
| --- | --- |
| Planned held-out folds | 25 |
| Successful held-out folds | 25/25 (100%) |
| Total true out-of-fold predictions | 11,325 |
| Pooled held-out agreement | 89.8% |
| Cohen's kappa | 0.847 |
| Mean held-out maximum PP | 0.838 |
| Held-out max PP ≥0.70 | 77.0% |
| Held-out max PP ≥0.90 | 46.9% |
| Mean class-shape correlation | 0.99985 |
| Minimum class-shape correlation | 0.99925 |
| Repeat-level overall agreement, range | 89.1%-90.8% |
| Same class in all 5 held-out predictions | 1,968/2,265 (86.9%) |
| Same class in ≥4 of 5 held-out predictions | 2,142/2,265 (94.6%) |
| Majority-vote agreement with full-cohort assignment | 2,071/2,265 (91.4%) |

*Internal validation used five repetitions of five-fold cross-validation in the NLR-eligible trajectory cohort (n=2,265). In each fold, the selected three-class quadratic GBTM was refitted in 80% of patients using the same GBTM specification as the primary analysis, and posterior class probabilities were calculated in the held-out 20%. Because latent-class numbering is arbitrary across refits, fold-specific classes were aligned to the full-cohort T1–T3 solution by minimizing differences in the five-window longitudinal NLR trajectory shapes, without using AKD outcomes. Held-out class agreement denotes agreement between the cross-validated assignment and the full-cohort assignment; class-specific agreement uses the full-cohort class as the reference. Mean held-out maximum PP is the mean maximum posterior class-membership probability. Shape correlation and RMSE compare each refitted class trajectory with its aligned full-cohort trajectory. Subject-level consistency is based on the five independent held-out classifications per patient. GBTM, group-based trajectory modeling; NLR, neutrophil-to-lymphocyte ratio; PP, posterior probability; RMSE, root mean square error.*

**Supplementary Table 18. Exploratory post-hoc thresholds for late postoperative NLR and AKD outcomes.**

| **Outcome** | **n / events** | **AUC (bootstrap 95% CI)** | **Apparent Youden threshold** | **Bootstrap median threshold (95% interval)** | **Apparent sensitivity / specificity** | **OOB median sensitivity / specificity** |
| --- | --- | --- | --- | --- | --- | --- |
| Overall AKD | 1,941 / 295 | 0.711 (0.680–0.743) | 7.70 | 7.91 (5.89–9.18) | 68.8% / 63.0% | 64.7% / 65.3% |
| Persistent AKD among patients with early AKI | 577 / 186 | 0.620 (0.572–0.665) | 8.37 | 8.94 (7.50–15.19) | 73.1% / 47.1% | 69.0% / 48.1% |
| Delayed AKD among patients without early AKI | 1,364 / 109 | 0.675 (0.621–0.722) | 5.89 | 5.42 (4.07–8.23) | 72.5% / 54.3% | 73.6% / 50.6% |

Late postoperative NLR was measured during 72–168 h after surgery. Persistent AKD was evaluated among patients with early postoperative AKI, whereas delayed AKD was evaluated among patients without early postoperative AKI. The apparent threshold maximized the Youden index. Threshold stability was assessed using 1,000 bootstrap resamples, with sensitivity and specificity evaluated in out-of-bag samples. These post-hoc analyses were exploratory and were not intended to define validated clinical cutoffs. AKD, acute kidney disease; AKI, acute kidney injury; AUC, area under the receiver operating characteristic curve; CI, confidence interval; NLR, neutrophil-to-lymphocyte ratio; OOB, out-of-bag.
